# A nomogram for predicting the likelihood of lymph node metastasis in early gastric patients

**DOI:** 10.1186/s12885-016-2132-5

**Published:** 2016-02-12

**Authors:** Zhixue Zheng, Yinan Zhang, Lianhai Zhang, Ziyu Li, Xiaojiang Wu, Yiqiang Liu, Zhaode Bu, Jiafu Ji

**Affiliations:** Department of Gastrointestinal Surgery, Key Laboratory of Carcinogenesis and Translational Research (Ministry of Education), Peking University Cancer Hospital & Institute, 52 Fu Cheng Road, Hai Dian District, 100142 Beijing, China; Department of Pathology, Key Laboratory of Carcinogenesis and Translational Research (Ministry of Education), Peking University Cancer Hospital & Institute, Beijing, China

**Keywords:** Early gastric cancer, Lymph node metastasis, Nomogram

## Abstract

**Background:**

Early gastric cancer is defined as a lesion confined to the mucosa or submucosa, regardless of the size or lymph node metastasis. Treatment methods include endoscopic mucosal resection or endoscopic submucosal dissection, wedge resection, laparoscopically assisted gastrectomy and open gastrectomy. Lymph node metastasis is strong related with survival and recurrence. Therefore, the likelihood of lymph node metastasis is one of the most important factors when determining the most appropriate treatment.

**Methods:**

We retrospectively analyzed 597 patients who underwent D2 gastrectomy for early gastric cancer. The relationship between lymph node metastasis and clinicopathological features was analyzed. Using multivariate logistic regression analyses, we created a nomogram to predict the lymph node metastasis probability for early gastric cancer. Receiver operating characteristic analyses was performed to assess the predictive value of the model.

**Results:**

In the present study, 58 (9.7 %) early gastric cancer patients were histologically shown to have lymph node metastasis. The multivariate logistic regression analysis demonstrated that the age at diagnosis, differentiation status, the presence of ulcers, lymphovascular invasion and depth of invasion were independent risk factors for lymph node metastasis in early gastric cancer. Additionally, the tumor macroscopic type, size and histology type significantly correlated with these important independent factors. We constructed a predictive nomogram with these factors for lymph node metastasis in early gastric cancer patients, and the discrimination was good with the AUC of 0.860 (95 % CI: 0.809–0.912).

**Conclusions:**

We developed an effective nomogram to predict the incidence of lymph node metastasis for early gastric cancer patients.

## Background

Gastric cancer is currently among the most common cancer worldwide and the second most common cause of cancer-related death [1–3]. Early gastric cancer (EGC) is defined as a lesion confined to the mucosa or submucosa, regardless of the size or the presence of regional lymph node metastasis [4–7]. Treatment options for EGC include endoscopic mucosal resection (EMR) or endoscopic submucosal dissection (ESD), wedge resection, laparoscopically assisted gastrectomy and open gastrectomy [8, 9]. Currently, although gastrectomy plus lymph node dissection is still the gold standard of treatment for EGCs, endoscopic surgical techniques have been widely accepted as an alternate treatment for EGC patients with the appropriate criteria to maintain the quality of life for a subgroup of EGC patients [7, 10–12]. Technically, endoscopic surgery is used to dissect the mucosal or the submucosal layer, with regional lymph nodes left untreated. Thus, identifying patients with a high risk of lymph node metastasis is crucially important for the application of endoscopic surgery.

The likelihood of lymph node metastasis is one of the most important factors to consider when determining the most appropriate treatment. The absence of lymph node metastasis is a prerequisite for EMR/ESD [12], which preserves gastric function and maintains quality of life by avoiding a radical gastrectomy. Endoscopic resection for EGC is currently the established choice of treatment in Korea and Japan because it is both minimally invasive and effective in the curative management of EGC [13, 14]. Endoscopic resection with curative intent is indicated only in tumors that fulfill the endoscopic resection criteria because these tumors rarely metastasize to lymph nodes [15]. Recently, based on a large-scale case series, expanded indications for endoscopic resection have been proposed because those tumors meeting the expanded criteria had no risk of lymph node metastasis [16]. Previous studies have suggested that the definite indications of endoscopic resection include differentiated adenocarcinoma, intramucosal cancer, a tumor size up to 20 mm and the absence of ulceration [17–19]. In the era of endoscopic resection, the accurate prediction of the risk of lymph node metastasis in EGC is crucial to select patients suitable for this procedure.

Nomograms have been developed to quantify risk factors of lymph node metastasis in several carcinomas [20, 21]. However, there is no predictive nomogram for the risk of lymph node metastasis in EGC, especially in the Eastern population, which has a high incidence of gastric cancer [22]. The aim of the present study was to identify risk factors for lymph node metastasis and construct a nomogram for patients with EGC to guide treatment.

## Methods

### Patients

Between December 1996 and December 2012, a total number of 597 patients who underwent surgery as an initial treatment for EGC were studied at the Peking University Cancer Hospital. All of the patients underwent surgery and achieved radical (R0) resection with a D2 lymph node dissection and were histologically proven primary EGC in accordance with the rules of the Japanese Gastric Cancer Association (JGCA) [23]. Patient characteristics, including age and sex, were collected, and information regarding tumor size, depth of invasion, macroscopic type, histology, and lymphovascular invasion were retrieved from medical records. The depth of tumor invasion was classified as mucosa or submucosa. The maximum diameter of the tumor was recorded as the tumor size. The carcinomas were classified into three macroscopic types: protruding type (type I); superficial type [type II, including elevated (IIa), flat type (IIb), and depressed type (IIc)]; and excavated type (III). Tumor differentiation was classified into two groups: the differentiated group, which included well or moderately differentiated adenocarcinomas, and the undifferentiated group, which included poorly or undifferentiated adenocarcinomas. Histologic type was classified according to the WHO classification for gastric cancer, including adenocarcinoma, signet-ring cell carcinoma, mucinous adenocarcinoma, etc. Lymph node involvement was classified according to the 7th edition of the Union for International Cancer Control (UICC) pN category: pN0, no metastasis; pN1,1–2 metastatic lymph nodes; pN2,3–6 metastatic lymph nodes; and pN3,≥7 metastatic lymph nodes. No patients received neoadjuvant therapy before surgery. This study was approved by the Institutional Review Board of the Peking University Cancer Hospital, and informed consent was obtained from all of the individuals.

### Statistical analysis and nomogram construction

All statistical analyses and graphics were performed using the SPSS 20.0 statistical package (SPSS Inc., Chicago, IL, USA) and R version 2.11.1 (The R Foundation for Statistical Computing, Vienna, Austria). The associations between lymph node metastasis and clinicopathological parameters were analyzed using the chi-square test (or Fisher’s exact test when appropriate). Continuous variables were transformed into an adequate form to fit the proportional hazards and linearity assumptions. Risk factors for lymph node metastasis were studied using a binary logistic regression modeling technique [24–26].

A nomogram was developed as a tool for identifying patients at risk for lymph node metastasis, and it provides a graphical representation of the factors that can be used to calculate the risk of lymph node metastasis for an individual patient by the points associated with each risk factor. The predictive accuracy of the model was graphically displayed using the receiver operating characteristic curve (ROC). The accuracy of the nomogram was then quantified using the area under the curve (AUC) for validation. An AUC of 1.0 indicates a perfect concordance, whereas an AUC of 0.5 indicates no relationship [27]. The ROC curve is a plot of sensitivity versus 1-specificity for different threshold probabilities of lymph node metastasis. The threshold probabilities are arbitrary cutoff points used to classify patients as lymph node metastasis and non-lymph node metastasis. The sensitivity is defined as the probability of the model predicting a patient will have lymph node metastasis, given that the patient has lymph node metastasis. The specificity is defined as the probability of the model predicting a patient will not have lymph node metastasis, given that the patient does not have lymph node metastasis. Calibration was performed for the constructed nomogram, and the nomogram was internally validated using 200 repetitions of bootstrap sample corrections. The probability of lymph node metastasis was estimated with 95 % confidence intervals (95 % CI) based on binominal distribution. P values of less than 0.05 were considered significant. Bootstrapping allows for the simulation of the performance of the nomogram if it was applied to future patients and provides an estimate of the average optimism of the AUC.

## Results

### The correlations between lymph node metastasis and the clinicopathological features of EGC patients

There were totally 597 patients involved in this study at Peking University Cancer Hospital, including 416 men and 181 women. 355 tumors were confined in the mucosal layer while 262 tumors invaded the submucosal layer. The average age was 58 years old (range, 24–82 years old) and the mean number of lymph nodes with metastases was 1 (range, 0–25) while the mean number of the total lymph node was 24 (range 9–60; IQR, P_25_:18, P_50_:23, P_75_:29). Lymph node metastasis was confirmed pathologically in 58 (9.7 %) patients. The number of patients of N0, N1, N2 and N3 stage were 539 (90.3 %), 39 (6.5 %), 10 (1.7 %), and 9 (1.5 %) respectively.

Lymph node metastasis was associated with age, macroscopic type, size, histology, differentiation, ulcer, lymphovascular invasion and depth of invasion (all *p* < 0.05). Patients younger than 50 years of age have a higher probability of lymph node metastasis than older patients (*p* = 0.024). The protruding and superficial-type carcinomas have a lower possibility of lymph node metastasis than the excavated and mixed type carcinomas (*p* < 0.001). Tumors larger than 2 cm were more likely to have lymph node metastases than smaller tumors (*p* = 0.004). Undifferentiated carcinomas and tumors with an ulcer or lymphovascular/submucosal invasion were associated with higher lymph node metastases (all *p* < 0.001). In gastric adenocarcinomas, the incidence of lymph node metastasis was lower than other pathological types (*p* = 0.001). There was no significant difference in gender or tumor location for lymph node metastasis (Table 1).

**Table 1:** Correlations between lymph node metastasis and clinicopathological features

Clinicopathological features	Lymph node metastasis		*p*
	Negative (*n* = 539)	Positive (*n* = 58)	
Gender			0.901
Male	376 (90.4 %)	40 (9.6 %)	
Female	163 (90.1 %)	18 (9.9 %)	
Age (year)			0.024
<50	108 (85.0 %)	19 (15.0 %)	
≥50	431 (91.7 %)	39 (8.3 %)	
Tumor location			0.179
Upper 1/3	89 (94.7 %)	5 (5.3 %)	
Middle 1/3	130 (92.9 %)	12 (8.5 %)	
Low 1/3	320 (87.9 %)	41 (11.4 %)	
Macroscopic type			<0.001
I/II	374 (94.4 %)	22 (5.6 %)	
III/Mixed	165 (82.1 %)	36 (17.9 %)	
Size (cm)			
<2.0	274 (93.8 %)	18 (6.2 %)	0.004
≥2.0	265 (86.9 %)	40 (13.1 %)	
<1.5	203 (96.7 %)	7 (3.3 %)	<0.001
≥1.5	336 (86.8 %)	51 (13.2 %)	
Histology			0.001
Adenocarcinoma	403 (92.6 %)	32 (7.4 %)	
Other types^a^	136 (84.0 %)	26 (16.0 %)	
Differentiation			<0.001
Differentiated	245 (96.1 %)	10 (3.9 %)	
Undifferentiated	294 (86.0 %)	48 (14.0 %)	
Ulcer			<0.001
Absent	463 (92.4 %)	38 (7.6 %)	
Present	76 (79.2 %)	20 (20.8 %)	
Lymphovascular invasion			<0.001
Absent	510 (95.0 %)	27 (5.0 %)	
Present	29 (48.3 %)	31 (51.7 %)	
Depth of invasion			<0.001
Mucosa	325 (97.0 %)	10 (3.0 %)	
Submucosa	214 (81.7 %)	48 (18.3 %)	

Other types^a^: signet-ring cell carcinoma, mucinous adenocarcinoma, etc

### The nomogram for the prediction of metastatic lymph nodes

We summarized the univariate and multivariate logistic regression analyses of lymph node metastasis (Table 2). The further multivariate logistic regression analysis showed that age (*p* = 0.028, RR 0.444, 95%CI: 0.215–0.916), differentiation (*p* = 0.002, RR 3.724, 95 % CI: 1.637–8.470), ulcer (*p* = 0.007, RR 2.710, 95 % CI: 1.310–5.606), lymphovascular invasion (*p* < 0.001, RR 13.703, 95 % CI: 6.515–28.822), and depth of invasion (*p* = 0.006, RR 3.013, 95 % CI: 1.369–6.631) were positively correlated with lymph node metastasis, indicating that these characteristics were independent risk factors of lymph node metastasis in EGC. Furthermore, we observed that the tumor macroscopic type, size, and histology were significantly correlated with the three most important independent factors (differentiation, lymphovascular invasion and depth of invasion; all *p* < 0.05; Table 3).

**Table 2:** Univariate and multivariate analysis of lymph node metastasis risk factors of early gastric cancer

Clinicopathological features	Univariate analysis	*p*	Multivariate analysis	*p*
	RR (95 % CI)		RR (95 % CI)	
Gender				
Male vs. Female	1.038 (0.578–1.865)	0.901		
Age (years)				
<50 vs. ≥50	0.514 (0.286–0.926)	0.027	0.444 (0.215–0.916)	0.028
Location				
Upper 1/3	1.000	0.190		
Middle 1/3	0.438 (0.168–1.143)	0.092		
Lower 1/3	0.720 (0.367–1.415)	0.341		
Macroscopic type				
I + II vs. III/Mixed	3.165 (1.758–5.696)	<0.001		
Size (cm)				
<2.0 vs. ≥2.0	2.298 (1.285–4.109)	0.005		
<1.5 vs. ≥1.5	4.402 (1.960–9.885)	<0.001		
Histology				
Adenocarcinoma vs. Other types^a^	2.408 (1.385–4.185)	0.002		
Differentiation				
Differentiated vs. Undifferentiated	4.000 (1.982–8.072)	<0.001	3.724 (1.637–8.470)	0.002
Ulcer				
Absent vs. Present	3.206 (1.772–5.803)	<0.001	2.710 (1.310–5.606)	0.007
Lymphovascular invasion				
Absent vs. Present	20.192 (10.675–38.191)	<0.001	13.703 (6.515–28.822)	<0.001
Depth of invasion				
Mucosa vs. Submucosa	7.290 (3.610–14.721)	<0.001	3.013 (1.369–6.631)	0.006

Other types^a^: signet-ring cell carcinoma, mucinous adenocarcinoma, etc

*RR* Relative risk

**Table 3:** Relationship between differentiation, depth of invasion and lymphovascular invasion with macroscopic type, size and histology

Clinicopathologic-al features	Differentiation		*p*	Lymphovascular invasion		*p*	Depth of invasion		*P*
	Differentiated (%)	Undifferentiated (%)		Absent (%)	Present (%)		Mucosa (%)	Submucosa (%)	
Macroscopic type			0.001			<0.001			<0.001
I/II	189 (47.7)	207 (52.3)		372 (93.9)	24 (6.1)		266 (67.2)	130 (32.8)	
III/Mixed	66 (32.8)	135 (67.2)		165 (82.1)	36 (17.9)		69 (34.3)	132 (65.7)	
Size (cm)			<0.001			<0.001			<0.001
<2.0	149 (51.0)	143 (49.0)		278 (95.2)	14 (4.8)		191 (65.4)	101 (34.6)	
≥2.0	106 (34.8)	199 (65.2)		259 (84.9)	46 (15.1)		144 (47.2)	161 (52.8)	
Histology			<0.001			0.255			0.724
Adenocarcinoma	241 (55.4)	194 (44.6)		395 (90.8)	40 (9.2)		246 (56.6)	189 (43.4)	
Other types^a^	14 (8.6)	148 (91.4)		142 (87.7)	20 (45.4)		89 (54.9)	73 (45.1)	

Other types^a^: signet-ring cell carcinoma, mucinous adenocarcinoma, etc

**Fig. 1:**
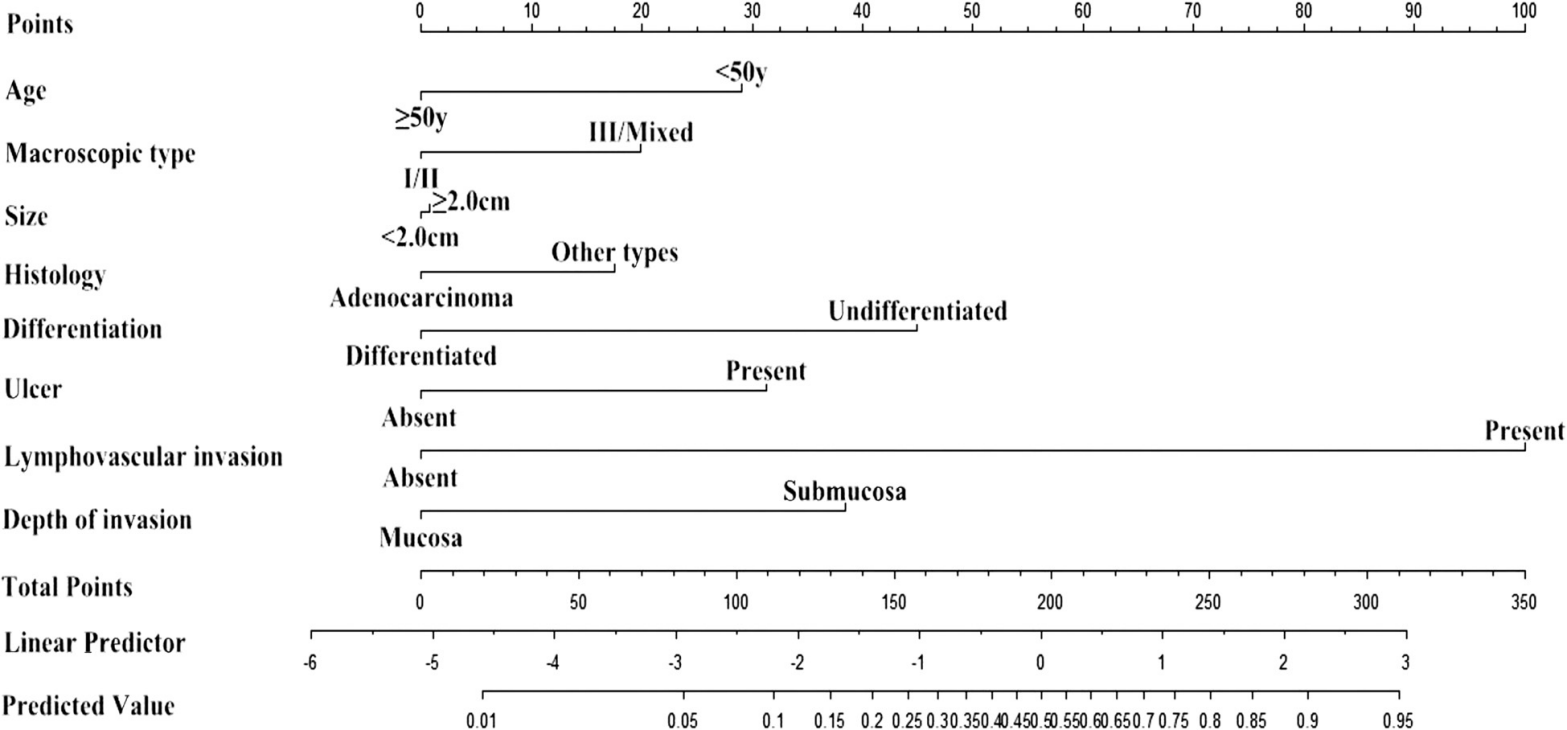
A nomogram predicting the probability of metastatic lymph node involvement for patients with early gastric cancer. The probability of metastatic lymph node involvement in early gastric cancer is calculated by drawing a line to the point on the axis for each of the following variables: age, macroscopic type, size, histology, differentiation, ulcer, lymphovascular invasion and depth of invasion. The points for each variable are summed and located on the total point line. Next, a vertical line is projected from the total point line to the predicted probability bottom scale to obtain the individual probability of metastatic lymph node involvement

**Fig. 2:**
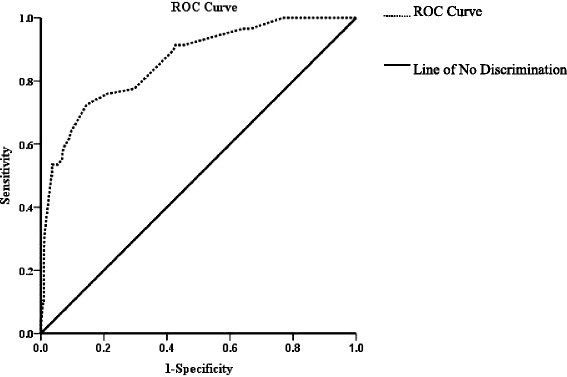
A receiver operating characteristics (ROC) curve of the multivariate logistic regression model for predicting lymph node metastasis for patients with early gastric cancer which had an AUC of 0.860 (95 % CI: 0.809–0.912), implying a good concordance

Thus, we chose these eight factors to develop a predictive nomogram for lymph node metastasis in EGC patients. The nomogram corresponding to the model including the possible factors that may affect the incidence of lymph node metastasis is show in Fig. 1. For each patient, points were assigned for each of these clinicopathological features (age, macroscopic type, size, histology, differentiation, ulcer, lymphovascular invasion, and depth of invasion), and a total score was calculated from the nomogram. The total points corresponded to a predicted metastatic lymph node metastasis probability. Furthermore, we developed a ROC curve to estimate the predictive accuracy of the model, which had an AUC of 0.860 (95 % CI: 0.809–0.912), implying a good concordance (Fig. 2).

## Discussion

Most surgeons consider D2 lymphadenectomy (dissection of all group I and group II lymph nodes) to be the standard and optimal surgical procedure for patients with EGC within the past ten years [28, 29]. The lymph node metastasis incidence of EGC is reported to be approximately 11 % to 18 %, and approximately 70 % to 80 % of patients will undergo overtreatment with D2 lymphadenectomy [29–32]. Recently, less invasive treatments have been performed for EGC, including endoscopic mucosal resection and endoscopic submucosal dissection [33, 34]. Because of recent advances in surgical instrumentation and techniques, laparoscopic procedures have also been suggested as an alternative minimally invasive treatment for EGC [35, 36]. For this purpose, we retrospectively analyzed 597 EGC patients based on clinical and routinely definitive pathological characteristics to investigate the evidence used for making medical decisions. Many cancer clinicians are increasingly becoming attracted to simple tools such as Nomograms to improve cancer treatment. In the present study, we have developed a nomogram that could predict the incidence of lymph node metastasis in EGC patients.

The lymph node metastasis incidence of all EGC patients was 9.7 % in the current study, 51.7 % in cancers with lymphovascular invasion, 20.8 % in cancers with an ulcer, 14.0 % in cancers with an undifferentiated histology, 18.3 % in submucosal cancers, 13.1 % in larger sized tumors (≥2.0 cm), 17.9 % in macroscopic type III/mixed, and 15.0 % in younger patients (<50 years old), which are similar to or lower than previous results [32, 38, 39]. In the multivariate analysis, the patients’ age at diagnosis, differentiation status, the presence of an ulcer, lymphovascular invasion and depth of invasion were independent factors for lymph node metastasis, and the presence of lymphovascular invasion was considered the most important predictor. Previous surveys have clarified the pathological characteristics of EGC with or without nodal metastases. Histologic ulceration of the tumor, larger size (≥20 mm) and submucosal penetration were independent risk factors for regional lymph node metastasis [39, 40]. Lymphovascular involvement and mixed histological type tumors have previously been reported as risk factors for nodal metastases [41, 42]. In general, the depth of tumor invasion reflects the progression of a tumor originating from the mucosal layer and is significantly associated with the presence of regional lymph node metastasis in EGC [18]. Our results were consistent with the above studies. Based on the previous studies and our results, the tumor size, macroscopic type and histology were considered to have significant clinical meaning among the risk factors of lymph node metastasis in EGC. Therefore, we chose these characteristic features in our nomogram for predicting the incidence of lymph node metastasis in EGC. This figure could generate estimates of the likelihood of metastatic lymph node involvement. Our nomogram appears to be simple and practical with a relatively high area under the ROC curve of 0.860, thus exhibiting a good performance related to a mean error that never exceeded 5 %. These findings support our selection of variables for determining suitable treatment.

The gold standard in the curative treatment of gastric cancer is a radical operation generally associated with D2 lymphadenectomy which has a high success incidence in early cases [43]. However, some complications and mortality are associated with this procedure that are not always necessary [44]. Certain groups of patients with EGC have a lower possibility of lymph node metastases, allowing less invasive treatment strategies to be adopted for these situations [38]. Endoscopic resection with curative intent is indicated only in tumors that fulfill the endoscopic resection criteria because these tumors rarely metastasize to lymph nodes [16]. These treatments preserve bodily functions and maintain quality of life. Our investigation has provided a good and helpful method to address this issue. For example, an undifferentiated submucosal gastric cancer patient with an ulcer and lymphovascular invasion, who is younger than 50 years of age, has more than an 80 % possibility of lymph node metastasis without considering other factors. This patient is suitable for a radical operation with lymphadenectomy or laparoscopic lymph node dissection following endoscopic dissection. In contrast, a patient with opposite characteristics, such as differentiated mucosal cancer, without an ulcer or lymphovascular invasion, and older than 50 years old, has almost no risk of lymph node metastasis with respect to other patients (less than 5 %), and should receive less invasive treatments. Therefore, we believe our nomogram will assist surgeons in selecting the appropriate treatment for patients with EGC with regard to the probability of lymph node metastasis.

To our knowledge, this is the first study providing a nomogram to predict the incidence of lymph node metastasis for EGC. The potential limitations of this study include the small cohort, and we should expand the sample size to improve the nomogram. Additionally, this is a single center retrospective study that needs further external validation with different populations. In this study, we did not use specific cutoff values of lymph node metastasis for different treatments with EGC. Despite these limitations, this nomogram offers an effective tool to predict the incidence of lymph node metastasis for EGC patients, with which we could select the appropriate treatments for patients.

## Conclusions

In conclusion, the present study constructed a nomogram to predict the probability of lymph node metastasis in EGC patients based on lymphovascular invasion, depth of invasion, differentiation, age, macroscopic type, size, and histology. This tool can assist clinicians and patients in quantifying the potential lymph node metastasis incidence to make surgical decisions. Certain patients are suitable for a radical operation or endoscopic dissection plus D2 lymphadenectomy, and some patients can be selected for only endoscopic dissection (ESD, EMR). For future studies, we should expand the sample size, add additional centers to prove this nomogram, and determine the cutoff value of the lymph node metastasis incidence for different treatments.

### Ethical statement

The study was approved by the institution Review Board of Peking University Cancer Hospital. All patients provided written informed consent.

## Abbreviations

EGC: early gastric cancer
EMR: endoscopic mucosal resection
ESD: endoscopic submucosal dissection
ROC: operating characteristic curve
AUC: area under the curve
CI: confidence intervals
